# Novel pulp capping material based on sodium trimetaphosphate: synthesis, characterization, and antimicrobial properties

**DOI:** 10.1590/1678-7757-2021-0483

**Published:** 2022-03-28

**Authors:** Nayara Rodrigues Sartori Franzin, Michela Melissa Duarte Seixas Sostena, Alailson Domingos dos Santos, Marcia Regina Moura, Emerson Rodrigues de Camargo, Thayse Yumi Hosida, Alberto Carlos Botazzo Delbem, João Carlos Silos Moraes

**Affiliations:** 1 Universidade Estadual Paulista Faculdade de Engenharia Ilha Solteira SP Brasil Universidade Estadual Paulista (UNESP), Faculdade de Engenharia, Ilha Solteira, SP, Brasil.; 2 Centro Universitário Santa Fé do Sul RS Brasil Centro Universitário (Unifunec), Santa Fé do Sul, RS, Brasil.; 3 Universidade Federal de São Carlos São Carlos SP Brasil Universidade Federal de São Carlos (UFSCar), São Carlos, SP, Brasil.; 4 Universidade Estadual Paulista Faculdade de Odontologia Araçatuba SP Brasil Universidade Estadual Paulista (UNESP), Faculdade de Odontologia, Araçatuba, SP, Brasil.

**Keywords:** Sodium trimetaphosphate, Nanoparticles, Compressive strength, Anti-bacterial agents, Chitosan, Titanium oxide

## Abstract

**Objectives:**

To evaluate the mechanical, physicochemical, and antimicrobial properties of four different formulations containing micro- or nanoparticles of sodium trimetaphosphate (mTMP and nTMP, respectively).

**Methodology:**

Four experimental groups were used in this investigation: two mTMP groups and two nTMP groups, each containing zirconium oxide (ZrO_2_), and solution containing either chitosan or titanium oxide (TiO_2_) nanoparticles (NPs). Setting time, compression resistance, and radiopacity were estimated. The agar diffusion test was used to assess the antimicrobial activity of the formulations against five different microbial strains: *Streptococcus mutans*, *Lactobacillus casei*, *Actinomyces israelii*, *Candida albicans*, and *Enterococcus faecalis*. Parametric and nonparametric tests were performed after evaluating homoscedasticity data (p<0.05).

**Results:**

From the properties evaluated, nTMP cements required less setting time and showed greater resistance to compression. Cements containing TiO_2_ showed greater radiopacity for both nTMP and mTMP. All four cement formulations showed antimicrobial activity against *S. mutans* and *L. casei*

**Conclusion:**

Formulations containing nTMP have shorter setting times and higher compressive strength, and those with TiO_2_ nanoparticles showed antimicrobial activities. Clinical relevance: The cement containing nTMP, ZrO_2_, and TiO_2_ could be an alternative material for protecting the pulp complex.

## Introduction

The most common causes of injuries to pulp tissue are deep cavities due to dental caries and dental trauma. If injured, the maintenance of pulp tissue integrity is achieved by pulp therapy. It aims to maintain, even if only partially, pulp vitality by eliminating bacteria from the dentin-pulp complex^1^ and preserve its functional and biological activities. Frequently used therapies are indirect and direct pulp capping. In direct pulp capping, the dental pulp is exposed, and a protective agent that induces the repair of hard tissue is used. In indirect pulp capping, as the pulp remains unexposed, a thin layer of material is applied on the dentin.^2^ To protect the pulp complex and preserve its vitality, an ideal pump capping material should be able to provide an optimal seal, minimize microleakage, and show low solubility, excellent bioactivity, dimensional stability, bactericidal properties, radiopacity, and high compression resistance.^3^ Different materials have been used in direct pulp capping, such as calcium hydroxide (Ca(OH)_2_) paste and mineral trioxide aggregate (MTA), because they offer excellent antimicrobial properties (pH ~ 12) and can promote the formation of a mineralized tissue barrier.^4-6^ Clinically, Ca(OH)_2_ paste is easy to handle and has an optimal setting time, but its high solubility produces a poor seal and it lacks adhesive properties.^7,8^ In contrast, MTA shows low solubility and excellent marginal sealing, but a lower antimicrobial activity than Ca(OH)_2_.^9,10^ Moreover, MTA has significant clinical disadvantages, such as long setting time,^11-13^ difficult handling, and high cost.^1,14-16^ Currently, there are various MTA-based products available on the market, with setting times ranging from minutes to hours, and some of them offer easy handling.

Sodium cyclophosphates, such as sodium trimetaphosphate (TMP), can preserve the stability and integrity of the enamel mineral surface,^17^ nucleate calcium ions,^18^ increase enamel remineralization,^18^ and obliterate dentinal tubules if associated with fluoride.^19^ Moreover, studies have shown that a reduction in TMP particle size increases their anti-caries potential.^20-24^ In view of the need for materials that show better mechanical, physicochemical, and antimicrobial properties, this study aimed to develop a cement containing micro- (mTMP) or nanoparticulate sodium trimetaphosphate (nTMP) and evaluate the effects of trimetaphosphate microparticles (mTMP) and nanoparticles (nTMP) on the physicomechanical, physicochemical, and antimicrobial properties of its four different formulations.

## Methodology

### Cement with micro- or nanoparticle TMP

Our novel cement consists of a powder containing mTMP or nTMP^20^, zirconium oxide (ZrO_2_) as a radiopacifier, an aqueous solution containing an emulsifier, and either titanium oxide (TiO_2_) or chitosan NPs. In the development of this material, depending on the proportion of the different components, the material either failed to set and/or expanded or contracted during hardening. Thus, at this stage of development, setting times and dimensional changes were considered. Four different formulations were prepared: (1) mTMP, ZrO_2_, and chitosan NPs (ZMC); (2) nTMP, ZrO_2_, and chitosan NPs (ZNC); (3) mTMP, ZrO_2_, and TiO_2_ NPs (ZMT); and (4) nTMP, ZrO_2_, and TiO_2_ NPs (ZNT). Percentages for each constituent of the powder formulations and the powder/liquid ratio (P/L) are shown in Table 1. As the consistency of a dental cement can vary according to its application, the powder/liquid ratio indicated in Table 1 gives the paste of each cement formulation a putty-like consistency, adequate for use as a direct pulp capping agent.

**Table 1:** Chemical composition of pulp-capping cements investigated in this study: ZMC, ZNC, ZMT, and ZNT

Sample	TMP (%)		ZrO_2_ (%)	P/L (mg/mL)	
	Micro	Nano		Chitosan	TiO_2_
ZMC	85		15	6.67	
ZNC		85	15	6.67	
ZMT	85		15		6.0
ZNT		85	15		6.0

### Measurements of mechanical, physicochemical, and antibacterial properties of the cement

#### Setting time

In this study, setting time was based on ANSI/ADA specification no. 57. In total, five samples were prepared for each cement formulation using a stainless-steel ring of 10 mm internal diameter and 2 mm thickness. The assembly, comprising the mold and test material, remained during the specified setting time in a cabinet at 37°C and with a relative humidity between 95 and 100%. Three minutes after the start of mixing, an indenter needle (Gillmore) with a mass of 100.0±0.5 g and a flat tip of 2.0±0.1 mm in diameter, was carefully lowered vertically onto the surface of the sample. This procedure was repeated every 30 seconds until the needle failed to make a complete circular indentation on the test material. The time period that elapsed from start of mixing to when the needle failed to make a complete circular indentation on the tested material surface determined the setting time.

### Compressive strength

For each cement formulation, 10 specimens were prepared using a cylindrical mold 4 mm in diameter and 6 mm in height. Each cement specimen was mixed on a glass plate with a steel spatula, and the obtained paste was inserted into the mold, which was supported under a microscope slide. Next, the assembly was stored for seven days in a cabinet at 37±1ºC and a relative humidity between 95 and 100%. After this storage period, the samples were removed from the molds for compressive strength measurements using a universal testing machine (EMIC - Equipamentos e Sistemas de Ensaios LTDA, model DL 3000), under a crosshead speed of 1 mm/min and a load cell of 200 kgf. The compressive strength (in MPa) was estimated by calculating the ratio of the failure load (in Newton) to the specimen cross-sectional area (in mm^2^).

### Radiopacity

Radiopacity tests were performed according to ANSI/ADA specification no. 57. For each cement formulation, three samples were prepared using a mold 1 mm thick and 10 mm in diameter. Each sample was positioned in the center of a dental intraoral X-ray sensor adjacent to an aluminum step wedge. The set was exposed to X-ray radiation emitted by an Saevo X-ray machine (Alliage S/A Indústria Médico Odontológica, Ribeirão Preto - SP, Brazil), model AXR, 1330 VA. During exposure, focal distance was fixed at 30 cm.

The radiographic density values (pixels/mm^2^) of the obtained images were estimated using Adobe Creative Cloud Photoshop. Thus, it was possible to compare the radiographic density of each cement formulation with the radiopacity of the different aluminum step wedge thicknesses. Radiopacity was assessed by the method proposed by Duarte, et al.^25^ (2009) which is expressed by the equation:


$$\frac{\left(\text{A}\times \text{e1}\right)}{\text{B}}+\text{e2}$$

in which *A* is the radiographic density of the material (RDM) minus the radiographic density of the step of the aluminum wedge immediately below the RDM; *B*, the radiographic density difference of the aluminum wedge steps immediately above and below the RDM; *e*1, the height of each step in the aluminum wedge device (1 mm); and *e*2, the thickness of the aluminum step immediately below the RDM.

### Antibacterial test

The antimicrobial activity of the cement samples was evaluated by the agar diffusion test, which was conducted according to Duque, et al.^26^ (2009). The strains used were *Streptococcus mutans* (ATCC 25175), *Lactobacillus casei* (ATCC 393), *Actinomyces israelii* (ATCC 12102), *Candida albicans* (ATCC 10231), and *Enterococcus faecalis* (ATCC 512299). Strains were subcultured on Brain Heart Infusion Agar (BHI; Difco, Le Point de Claix, France) and incubated at 37°C for 48 hours under anaerobic conditions for *S. mutans*, *L. casei*, *A. israelii*, and *E. faecalis* and under aerobic conditions for *C. albicans*. Subsequently, five colonies of each strain were individually inserted into a BHI broth for 18–24 hours at 37°C. The 300 μL aliquot of each bacterial suspension (0.6 optical density and 550 nm absorbance) was homogenized with 15 mL of BHI agar at 45°C. After the culture medium solidified, 10 wells (4 mm each in diameter) were made in each plate and sequentially filled with one of the experimental materials at three different times. Materials were prepared and inserted into the wells. As a control for the experiment, 5 μL of aqueous 0.2% chlorhexidine (CHX) was used.^27^

### Statistical analysis

Statistical analysis was performed with the SigmaPlot software (Systat Inc, San Jose, CA, USA), version 12.0. Significance level was set at p<0.05. Setting time, compressive strength, and radiopacity data showed a normal (Shapiro-Wilk) and homogeneous (Cochran test) distribution and were submitted to one-way variance analysis, followed by a Student-Newman-Keuls test. Data from antimicrobial tests showed a heterogeneous distribution and were submitted to a Kruskal-Wallis test, followed by a Student-Newman-Keuls test.

## Results

Table 2 shows the values obtained for setting time. Using nTMP, instead of mTMP, in the cement formulation reduced setting times in a little more than 50%. The TiO_2_ groups showed a shorter setting time than the chitosan groups. The results obtained in the compressive strength test showed greater resistance to compression in groups containing nTMP with either TiO_2_ or chitosan NPs (Table 2). Groups with chitosan NPs showed higher compressive strength values (MPa) than those with TiO_2_, regardless of particle size. The cement with the greatest compressive strength was ZNC (p<0.05). We found statistically significant differences in the compressive strength of the groups analyzed, whose mean values ranged from 2.24±0.41 to 5.99±1.04 MPa.

**Table 2:** Mean values (SD) of setting time, compressive strength, and radiopacity of the four different cement compositions

	Analyses		
Materials	Setting time	Compressive strength	Radiopacity
	(min)	(MPa)	(mm Al)
ZMT	12.97^c^	2.24^a^	3.42^a^
	(0.15)	(0.41)	(0.13)
ZNT	5.75^a^	3.86^c^	3.20^a^
	(0.13)	(0.91)	(0.10)
ZMC	13.34^d^	2.81^b^	3.53^a^
	(0.39)	(0.40)	(0.35)
ZNC	6.22^b^	5.99^d^	3.47^a^
	(0.07)	(1.04)	(0.26)

Means followed by the different letter indicate significant difference among the materials for each analysis (ANOVA, Student-Newman-Keuls test, p<0.05).

Table 2 summarizes the results of the analysis performed on the radiographic images. All four cement formulations satisfy the minimum conditions required in the ANSI/ADA specification and we found no statistically significant difference (p=0.409) between the analyzed groups.

**Figure 1:**
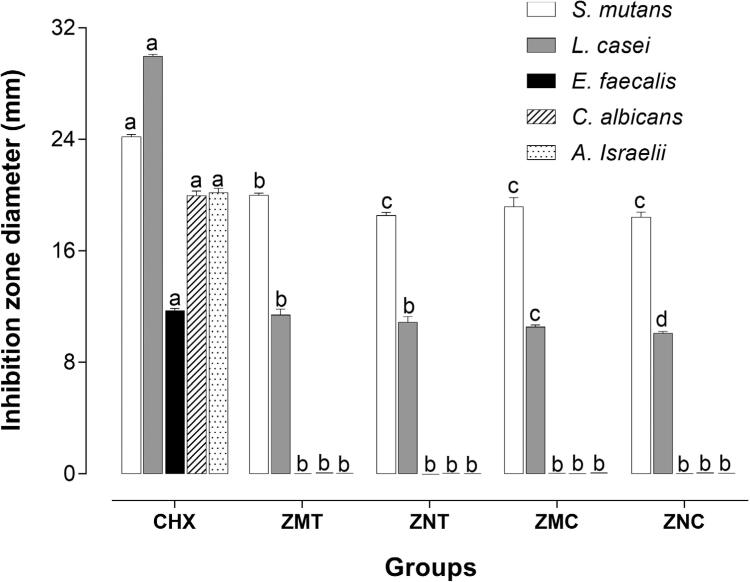
Mean values (SD) of inhibition zone diameters according to the microorganisms and the different cement formulations. Different lowercase letters show statistical differences between the groups for each bacterial species (Kruskal-Wallis, Student-Newman-Keuls test, p<0.05)

The groups with TiO_2_, ZMT (19.98±0.45 mm for *S. mutans* and 11.82±1.24 mm for *L. casei*) and ZNT (18.63±0.7 mm for *S. mutans* and 11.70±1.28 mm for *L. casei*), yielded better results for the diameter of inhibition zone than the cements with chitosan (Figure 1), ZMC (18.12±1.94 mm for *S. mutans* and 10.64±0.44 mm for *L. casei*) and ZNC (18.57±1.13 mm for *S. mutans* and 10.17±0.4 mm for *L. casei*). None of the tested groups showed antimicrobial action against *A. israelii*, *C. albicans* and *E. faecalis*. However, we found that all four cement formulations had smaller inhibition zone diameters than CHX.

## Discussion

Ideally, the application of a material in pulp capping procedures requires adequate mechanical, physicochemical, and antimicrobial properties to eliminate residual bacteria in decayed dentin, induce the regeneration of lost tissue, and prevent secondary infection and the possibility of pulp damage. In this study, incorporating TMP in different particle sizes affected the mechanical, physicochemical, and antimicrobial properties of the cements.

As part of the continuous evolution in conservative dentistry, there has been motivation for research to further investigate the possibilities of inducing repair and regeneration of lost hard dental tissue. Phosphates, such as sodium trimetaphosphate, can attract calcium ions, and, therefore, act as nucleating agents for apatite crystals.^28,29^ In particular, this process can induce mineralization of dentinal tissue,^30,31^ in addition to stimulating dental tissue repair and regeneration.

Ideal physicochemical properties are essential for a dental material to be suitable for clinical use. These physicochemical attributes will generally dictate the clinical indications of the material. Setting time is an important parameter for dental healthcare professionals since it represents the time interval available for the clinical procedure to be performed. There is a statistically significant difference in the mean setting time values among treatment groups (p<0.001). Using nanoparticulate TMP in cement formulations led to a reduction of 55.7 and 53.4% in the setting times of the cements with TiO_2_ and chitosan NPs, respectively. It is well known that particle size affects certain physical and chemical properties of cements.^32,33^ Specifically, the finer the cement particles are, the larger the surface contact area with the aqueous medium, increasing hydration efficiency and, consequently, accelerating the chemical reactions that occur during hardening. The setting time values obtained for the four cement formulations are suitable for application as pulp cappers when compared, for example, with the commercial MTA Repair HP (Angelus, Brazil) and Dycal (Dentsply, USA) cements. Both cements are indicated for pulp capping, according to information contained in the package insert of the product. MTA Repair HP has a setting time of 13±2 min and Dycal is close to 1 min.^34,35^ In comparison, ZNC and ZNT cements have setting times between these two commercial cements.

TMP powder particle size influenced the compressive strength of each of the four cements researched. Moreover, ZrO_2_ association may contribute to low compressive strength values. Studies show that the compressive strength of Portland and glass ionomer cements decreases when combined with zirconium oxide.^36,37^ Thus, zirconium oxide may have contributed to the low values observed for the compressive strength of the cements tested in this study. However, further research is necessary to assess the influence of the ZrO_2_ component on this mechanical property. Despite the possible negative influence of this compound in the compression resistance test, all four cement formulations satisfy the minimum conditions required in the ANSI/ADA specification for radiopacity. It is important to emphasize that radiopacity is a useful resource for identifying recurrent caries and evaluating endodontic repairs.

In this study, the new cements based on nTMP/mTMP, ZrO_2_, and chitosan or TiO_2_ showed antimicrobial activity for *S. mutans* and *L. casei*. These results are possibly associated with the fact that for both species of gram-positive bacteria, the chitosan action mode is an electrostatic interaction in which the positively charged chitosan is attracted to the negatively charged cell membrane, changing cell wall permeability. This change can cause the rupture of the membrane, leakage of protein components and other intracellular components, and, consequently, cell death. Regarding the action mechanism of TiO_2_ nanoparticles against anaerobic gram-positive bacteria, TiO_2_ exhibits toxicity in the presence of O_2_, in which lipid peroxidation causes membrane damage.^38^ Moreover, this results in the production of reactive oxygen species (ROS) which are responsible for the destruction of membrane layers.^39^ Although studies show that TMP, associated with fluoride, reduces the mineral loss of dental structures (confirmed by a clinical study in children^40^) and that a reduction in particle size has been shown to increase its anti-caries potential, TMP lacks antimicrobial or antifungal activity.^41^ However, since the phosphate can bind to the essential metal cations Ca^2+^ and Mg^2+^ present in the bacterial cell wall,^42^ TMP could potentiate the antimicrobial effects of chitosan and TiO_2_ nanoparticles, with the expectation of better results in nanoparticulate form. However, study results have shown no dependence on particle size.^22^

Regarding the other microorganisms evaluated, *A. israelii*, *E. faecalis*, and *C. albicans*, the groups evaluated showed no antimicrobial action. The explanation for the non-inhibition of both bactericidal agents is related to numerous factors arising from the characteristics and structures of the bacteria. *A. israelii*, despite being classified as gram-positive, showed resistance to antimicrobial action due to its morphology and resistance to oxygen, which proved that this microorganism shows irregular filaments. This difference in its filaments increases the structural strength of the bacterium and contributes to its resistance. *E. faecalis* has proteins on its surface that differ from other bacteria and give it the strength to survive in different environments and against different drugs and bactericidal agents.^43^ In regard to *C. albicans*, non-inhibition may be due to the more complex cell structure of yeasts and hyphae compared to bacteria, which possibly hampers the effects of chitosan and TiO_2_ nanoparticles.

This investigation indicates that the formulation containing nTMP, ZrO2, and TiO_2_ nanoparticles showed the best results due to its lowest setting time, high compressive strength, and antimicrobial activity in relation to *S. mutans*, which was very close to the ZMT formulation.
